# Lectures on Dental Physiology and Surgery, Delivered at the Middlesex Hospital School

**Published:** 1846-12

**Authors:** John Tomes

**Affiliations:** Surgeon Dentist to the Hospital.


THE AMERICAN

### Journal of Dental Science.

Vol. VII.] DECEMBER, 1846. [No. 2.

### ARTICLE I.

*A Course of Lectures on Dental Physiology and Surgery, delivered at the Middlesex Hospital School, Session* 1845–46. Expressly corrected for this Periodical by the Author, JOHN TOMES, ESQ., Surgeon Dentist to the Hospital.

### LECTURE VI.

ERUPTION OF THE TEMPORARY TEETH—GROWTH OF THE JAWS—ERUPTION OF THE PERMANENT TEETH—THE TEETH AN INDEX TO THE AGE WHEN BETWEEN SEVEN AND THIRTEEN YEARS—THIRD SET OF TEETH—SUPERNUMERARY TEETH—BLOOD-STAINED AND JAUNDICED TEETH—HEREDITARY CHARACTER OF TEETH—FORM OF THE TEETH AND PALATE AS NECESSARY TO VOICE AND SPEECH.

GENTLEMEN:—At the conclusion of our last lecture, we were considering the parts concerned in the development of the dental tissues. I shall again draw your attention to the subject of development for a few moments, and then pass to the eruption of the teeth.

Much has been written upon the membranes of the teeth. Mr. Bell divides them into deciduous and persistent. The deciduous membranes are two only; the two composing the dental sac. The persistent membranes are three in number: 1st, the lining membrane of the dental cavity, and the secreting membrane of the tooth substance; 2d, the periosteum of the root; and 3d, the periosteum of the alveoli. The periosteum of the maxillary bones was considered as the origin of the three persistent membranes. These views, which for many years were received as established, are now superseded by those advanced by Mr. Goodsir; which, I believe, have, to a great extent, been confirmed by subsequent investigations made by other anatomists.

In a former lecture, when tracing the analogy between the teeth and the mandible of birds and tortoises, I mentioned that there was considerable similarity in the mode of development. Mr. Owen has found that the bill of the tortoise is developed in separate parts, similar in form to the papilla of the teeth. The parts, at first separate, become confluent as the process of formation advances, so that at birth the papillary origin is no longer traceable.

### *Eruption of the Temporary Teeth.*

The next subject for our consideration will be the eruption of the temporary teeth. Teething, as this process of nature is not uncommonly denominated, is alike interesting to the physiologist and to the medical practitioner. To the one, the absorption of the gum and dental sac, in making way for the growing tooth, forms an interesting subject for observation; to the other, the time and the signs which precede and accompany the eruptions of the teeth, are matters of interest, and an acquaintance with which enables the practitioner to answer the oft-repeated questions, "When should the teeth be cut? Why are they not? Will any harm come as a consequence of the late appearance of the teeth?" And a thousand other such questions, suggested to the minds of anxious parents.

We have seen that as early as the sixth week of intra-uterine life, dental development commences. From that period the growth of the dental apparatus, in common with the other organs of the body, steadily progresses. At a definite period of infancy, the teeth, unlike the other organs of the body, are perfected, and grow no more. Previous to the completion of the fang, the crown, which is already perfected, appears through the gum, after which the root is rapidly developed. But before the body of the tooth can assume its ultimate position in the alveolar arch, the super-imposed gum must be removed. This action proceeds gradually, and without any manifest inconvenience to the healthy infant, until the mucous membrane alone remains over the surface of the crown of the tooth, when slight symptoms, characteristic of local irritation manifest themselves. These are not, however, invariably present; for it sometimes happens that teeth are cut without any attendant irritation, and their coming has been unobserved, unless the tooth has passed through the gum. But the absorption of the gum is called into action; whether by the pressure of the tooth, or by the laws regulating development, and thus preparing a passage for the tooth, so soon as space is required, is a point as yet undetermined. Prior to the appearance of a tooth, the super-imposed gum increases in breadth, becomes turgid, and feels hot to the touch. Accompanying these symptoms, there is an increased flow of saliva, which constantly escapes from the mouth; the child thrusts whatever may be placed in its hands to the mouth, and frequently bursts out in a fit of crying, when any foreign substance has come in contact with the gum. The breast is often suddenly relinquished, and again seized; in a word, we have just such symptoms as we should expect would arise from an irritable and sore gum. The child is more fretful than usual, and has sudden fits of crying, starts in its sleep, which is liable to be disturbed. The cheeks are occasionally flushed, the stomach and bowels are likely to become deranged also, as manifested by ejection of the food, or diarrhœa. A dry hard cough sometimes attends the eruption of each tooth. Even these symptoms of teething are, however, in some instances absent; a condition we should naturally expect, when it is considered that dentition, though frequently attended with suffering, is in itself a healthy action, and therefore, when accompanied with perfect health throughout the body, would necessarily be unattended with suffering. Dissection has shown that the papillæ of the milk teeth are not developed all at one time, but in succession, one following the other. From this, we might suppose that the teeth would appear through the gum in the same order in which the papillæ appeared in the maxillæ. The temporary teeth do appear in pairs, but not in the same order as the papillæ have been seen to take their respective places in the primary dental groove.

About the sixth, seventh or eighth month after birth, the central incisors of the under jaw appear through the gum. These are, in a week or two, succeeded by the corresponding teeth of the upper jaw.

In a month or six weeks after the eruption of the central incisors, we may expect the lateral incisors to make their appearance; those of the under jaw being evolved first. About the twelfth or fourteenth month, the anterior molars of the under jaw are cut, and shortly afterwards those of the upper maxilla. The canines appear between the sixteenth and twentieth; and between the twentieth and thirtieth month the second milk molars pass through the gum. Thus, according to this statement, the twenty milk teeth are complete by the thirtieth month. You must not, however, expect to find in all instances this regularity either as to the time or order of the eruption of the teeth.

Authors do not, however, agree in their statements as to the time at which the various teeth are cut. I shall therefore direct your attention to the table, in which is arranged, under separate heads, the time at which each tooth appears, as stated by some of our best writers upon dental surgery. I premise, that the following are stated only as the average periods at which the teeth appear.

| Authors. | Cent'l incisors. | Lat'l incisors. | Canines. | First molar. | Second molar. |
|---|---|---|---|---|---|
| | Months. | Months. | Months. | Months. | Months. |
| Fox,.......... | 6, 7, 8, extreme cases 4 to 13 | 7, 8, or 9 | 17 to 18 | 14 to 16 | 24 to 30 |
| Hunter,....... | 7, 8, 9 | 7, 8, or 9 | 20 to 24 | 20 to 24 | 20 to 24 |
| Bell,.......... | 5 to 8 | 7 to 10 | 14 to 20 | 12 to 16 | 18 to 36 |
| Dr. Ashburner, | 7th for the lower jaw, 8th for the upper. | 9th for the upper, 10th for the lower teeth. | 16, 17, 18, 19, or 20 | 12 to 14 | 22 to 30 |

The temporary teeth are very rarely subject to irregularity either in position, number or form. There are, however, cases recorded, in which the usual order was departed from. Dr. Brown, in the American Journal of Dental Surgery, mentions an instance which came under his own observation, in which the central incisors were through at birth. This occurred in a first child, and the teeth were extracted. Afterwards two other children were born, each with the central incisors of the under jaw through. The three children were girls, and in the two last the teeth were allowed to remain.

Many other instances have been described, in which one or more central incisors were through at birth. Duval mentions two cases, a father and daughter: in each an incisor of the lower jaw was present at birth. The same author relates a case in which the central incisors never appeared. Fouchard speaks of a child who, at six years of age, had only a few front teeth.

There are two cases recorded, one by Boxalli, another by M. Baumes, in which the patient reached old age without a single tooth ever having appeared.

I am not aware that there is any well attested case of malformation of a temporary tooth. We, however, sometimes see a condition somewhat analogous to malformation, in the lateral union of two teeth. Three cases of this kind have come under my observation within the last two years, in two of which the central incisors of the under jaw were laterally united, and in the third a lateral incisor and canine of the under jaw were united. The line of junction was marked by a shallow groove coated with enamel. Fouchard gives two cases, in which there was union between the lateral incisor and the canine; in one instance in the teeth of the upper jaw; in the other in the teeth of the under jaw.

At present I have called your attention to dentition as it occurs in healthy children, unattended by any unfavorable symptoms; in a future lecture, the subject of interrupted dentition, with the concomitant disorders, will come under our consideration.

We have now traced the temporary teeth, from their first appearance in the maxilla to their completion, when they become efficient organs of mastication and articulation. This state they retain, until, with the lapse of time, the jaw increases in size, and stronger organs of mastication become necessary; nature then effects their removal by withdrawing their support, in absorption of the fangs; the crown, then having no hold on the jaw, falls out. The spaces thus left are speedily filled by the permanent teeth, which rise up from within the alveolus. We are accustomed to speak of the removal of the roots of temporary teeth as effected by absorption; however, there is but little known of the nature of this process. All we really know is, that the root is removed gradually, that the process commences on the surface of the root, and generally upon one spot only, from which it extends gradually till all the root is removed. Many writers, assuming that the tooth is absorbed by the adjoining tissue, attempt to explain how the action is produced; some believing that it arises from pressure on the temporary by the advancing permanent tooth; others, again, suppose that pressure has no such effect. The process of absorption of the fang of a temporary tooth usually commences on the surface near the apex, and produces, as its first effect, a small hollow; from this point the action spreads till the end of the fang has been exhausted; it then proceeds towards the crown, until arrested by the edge of the enamel. The tooth, having lost all hold upon the gum, falls out. We then see that even the crown has not wholly escaped the devastating force, for it is found to be hollowed out from within. In some instances, absorption commences upon two points of the fang, and spreads from each. Such is the process when effected under the most favorable circumstances. But it not unfrequently happens that absorption commences high up the fang, producing partial removal only. In the meanwhile, the permanent tooth advances, and occupies the space formed by the removal of the one half of the fang of the milk tooth. In such instances, the absorption no longer continues in the remaining half of the temporary fang; the action is arrested, and the tooth is retained, slightly displaced itself, and slightly displacing the permanent tooth. This condition is more commonly met with in the canine teeth, though by no means uncommon in the incisors of the upper jaw. In the milk molars, absorption goes on in the several fangs at the same time. How the absorption of the fangs of the milk teeth is effected, what is the nature of the process, or whether the process is really an active force exercised upon the tooth by the adjoining parts, or inherent in the tooth, remains at present to be determined. It is certain that if a milk tooth be dead, the fang is not absorbed. From this it may, I think, be fairly assumed, that it is necessary that there should be vitality in the tooth for the absorption to go on. On the other hand, I have repeatedly observed, that the surface of the periosteum opposed to the wasting surface of the fang is highly vascular, and closely applied though inadherent to that surface. On removing a milk tooth, where the whole of the crown has been removed, we find the crown excavated, and the excavation occupied by an unattached vascular papilla.

From these facts, we are led to conclude, that not only must a tooth possess vitality, but also that the surface where absorption is to proceed, must be unattached to, but placed in contact with a highly vascular structure.*

Though we seem to possess some tolerably definite knowledge as to how the absorption of fluid matter is effected in organised bodies, namely, by imbibition, yet at present, information is wanted as to the manner in which a solid living tissue is removed; and until this is gained, our information on the subject of dental absorption will remain indefinite.

### *Eruption of the Permanent Teeth.*

The period destined by nature for the existence of the milk teeth having passed, and their removal having been effected, the succeeding permanent teeth take their place in the jaws. This period varies in the different teeth, and is subject to some little variety in every individual.

On entering upon the subject of the second dentition, we must bear in mind that the molar teeth are not preceded by temporary teeth, but are cut like the teeth of the first dentition,

* Since writing this lecture, I have seen a description given by Dr. Ashburner, in his work on Dentition, in which the vascular structure is considered as a special organ for absorption.

each molar passing through the gum so soon as, by the growth of the jaw, sufficient alveolar surface is afforded. The second dentition, in the majority of cases, commences about the seventh year, and proceeds in the following order:

| | |
|---|---|
| First molar, - - - - | 7 years. |
| Central incisor, - - - | 8 " |
| Lateral incisor, - - - | 9 " |
| First bicuspis, - - - | 10 " |
| Second bicuspis, - - - | 11 " |
| Cuspidatus, - - - | 12 " |
| Second molar, - - - - | 13 " |

The teeth of the lower jaw are commonly somewhat earlier by a month or two than those of the upper jaw.

At the sixth year the jaw contains both the temporary and the permanent teeth; the former in a perfect state, the latter partly formed.

In describing the development of the teeth, the relative position of the two sets was noted; but I will again call your attention to them at this particular age, since, by a knowledge of the relation of the two sets, we are enabled to understand and to treat the irregularities which sometimes occur in permanent teeth. To use the words of Mr. Bell: "The jaws, at from six to seven years of age, contain no less than forty-eight teeth; namely, twenty deciduous, the whole of which are perfected, and twenty-eight permanent, in different degrees of development, within the bones.

The following is the relative position of the two sets at this period. The permanent central incisor of the lower jaw is placed immediately beneath the temporary, with its point directed a little backwards, behind the partially absorbed root of the latter. The lateral incisor, not yet so far advanced, is placed deeper in the jaw, and instead of being immediately beneath the temporary, is situated with its point between the roots of this and the cuspidatus. The permanent cuspidatus is still very deeply imbedded in the bone, with its point rising between the roots of the temporary cuspidatus and the first temporary molaris. The two spreading roots of the latter encompass, as it were, within their space, the first bicuspis, and those of the second temporary molar in like manner the second bicuspis.

"Nearly a similar arrrangement is found to exist in the upper jaw, excepting that the teeth are altogether more crowded. The lateral incisor is placed farther back, particularly its point; the cuspidatus is directed more outwards, as well as the bicuspides."

It has been generally supposed that the permanent teeth are very irregular as to the time of their appearance; so much so, indeed, that no estimate of the age of a child could be formed by the number of permanent teeth present. Such, however, is not the case. Mr. Saunders, with great success, investigated the subject statistically, and has been led to the following results:—First, that at nine years of age the first permanent molars are developed. Second, that at the age of thirteen the whole of the permanent teeth, excepting the wisdom teeth, are present. In Mr. Saunders' monograph, entitled, "The Teeth as a Test of Age," is a full account of his researches. He concludes with the following paragraph:

"Thus, then, it appears, that of 708 children of nine years of age, 389 would have been pronounced, on an application of this test, to be near the completion of the ninth year; that is, they presented the full development of that age. But on the principle already stated, that of reckoning the fourth tooth as present when the three are fully developed, a still larger majority will be obtained, and, instead of 389, the proportion will be as follows:—Of 708 children, no less a number than 530 will be fully nine years of age. What, then, are the deviations in the remaining 178? They are the following:—126 would be pronounced eight years and six months, and the remaining 52 eight years of age; so that the extreme deviations are only twelve months, and these only in the inconsiderable proportion (when compared with the results obtained by other critera,) of 52 in 708.

"Again, of 338 children under thirteen years of age, no less than 294 might have been pronounced with confidence to be of that age. The remaining 44 would have been considered as follows:—36 in their thirteenth, and 8 near the completion of their twelfth year."

It would seem, from Mr. Saunders' statements, that the intermediate ages between nine and thirteen—indeed from seven to thirteen—may be estimated by examining the teeth. I am not aware that these assertions are backed by extended statistical research.

I have been, during the past two years, engaged in collecting statistical facts upon the subject, but at present I have been unable to put them into definite form ; however, I am prepared to corroborate the general correctness of Mr. Saunders' views.

### *Growth of the Jaws.*

The maxillæ, in their growth, present the peculiarity of increasing at the posterior part only. After the eruption of the second milk molars, we find them placed at the base of the coronoid process of the lower, and at the posterior edge of the upper jaw. At the age of seven, we find, on the contrary, the milk molars situated considerably in advance of these points. The jaws have, in fact, grown at these points; that is, anterior to the coronoid process in the under jaw, and the tuberosity in the upper jaw. At the same time that growth has proceeded rapidly at the posterior division, no increase whatever has taken place anterior to the last milk molar, excepting some slight addition in depth. The alveolar space found at the seventh year is, about that time, wholly occupied by the first permanent molar, which, when cut, lies close to the base of the before-named process. The maxillæ still grows, not, however, at the points between the coronoid process in the tuberosity and the last milk molar, but between the newly-developed first permanent molar and these processes. So that a second space is formed in the jaw, which is, when sufficient, occupied by the development of the second permanent molar. The growth continuing posterior to the second molar, a third space is formed, which is filled by the wisdom tooth. The jaws then cease to grow, and change only after the removal of the teeth.

Some few cases are recorded, in which the permanent teeth were said to be removed, and a third set to have taken their place. I have not, in my own practice, seen any confirmation of these statements, neither are dentists generally disposed to give much credit to the assertion. It would seem improbable that a third set of teeth should be formed, yet it is by no means impossible. We see nature depart from her ordinary law in producing irregularity in various parts of the body. I do not know, therefore, why we should regard the production of a third set of teeth as absolutely impossible. The point, however, is not likely to be set at rest, for the statement of patients cannot be depended on, as they are unable to distinguish between the temporary and permanent teeth, and dentists are not themselves likely to see, from infancy to old age, a patient subject to this peculiarity.

### *Supernumerary Teeth.*

Thirty-two is the number of the permanent teeth; but supernumerary teeth are not very uncommon. When found, they are mostly of a conical form, single-fanged, and placed between the other teeth. I have seen a case in which a supernumerary tooth had grown between and behind the central incisors of the upper jaw. Sometimes they are placed between the molars: I believe they are far more common in the upper than in the lower jaw. When occurring between the molar teeth, the supernumerary tooth looks in form, and sometimes in position, as though it had been formed by the disunion of one of the cusps and roots of the contiguous tooth during the earlier stages of its development.

### *Hereditary Character of the Teeth.*

It is a well-attested fact, that the properties of the parent are, to a considerable extent, transmitted to the offspring. In no part of the body is this hereditary character more frequently displayed than in the teeth, and especially as regards the external form. If the teeth of the parents be strong and well formed, we may expect to find similar teeth in the child—unless, indeed, the child be unhealthy. Peculiarities in color and in position are often transmitted. The predisposition to early decay often passes from the parent to the child. It does not, however, follow that the hereditary peculiarity in dental formation will in all cases appear; on the contrary, we sometimes see cases in which the reverse has manifestly occurred. Again, a peculiarity may not reappear in the first or even the second generation, but show itself in the third remove from the original parent. These facts are worth bearing in mind, as they often influence the treatment of the anormal conditions of the teeth. Not that the fact of a malady having occurred in the parent of a patient will of itself indicate a peculiar treatment; but you will probably learn how the remedy was, or was not, effected by the treatment adopted in this previous instance. This knowledge you will find especially useful when similar cases have occurred in the children of one family.

It has been observed, in persons who have died from suffocation, that the teeth are in some cases stained red. This state, before the structure of the teeth was understood, was considered to be a proof of their vascularity. It was supposed that the blood imparting the color was contained in vessels, which, in these cases, were gorged by the mode of death. This, I need not tell you, is an incorrect hypothesis: vessels, when they do exist in the substance of the tooth, are never present in such number as to give a red color to the dental tissues. We shall find the more correct explanation, by considering the state of the blood in asphyxia. The coloring matter of normal blood resides entirely in the globules. The liquor sanguinis is perfectly colorless. But under certain circumstances the colored globules decompose, or are dissolved in the liquor sanguinis, which then becomes a deep red. The tubuli of the teeth, though too small to admit the red globules of the blood, freely admit the liquor sanguinis; and if this be colored red, the tooth itself will necessarily take the same hue.

In persons suffering from jaundice, the teeth become tinged. In some few instances they take a deep yellow, almost equalling in intensity the color of the skin, but in the majority of cases they assume only a faint tinge of yellow.

In taking a retrospective view of the anatomy and physiology of the teeth, we are forcibly struck with the adaptation of these organs to their peculiar functions. Early in life, when the jaws are small and comparatively powerless, the small and more delicate milk teeth are provided by nature to perform the necessary office of mastication. When the body has increased in size, and the jaws have become larger and more powerful, the smaller teeth are, by a natural process, removed, and a stronger and more numerous set is developed, with which we are enabled to masticate the more solid food required for the sustenance of the adult.

Like the bones generally, the teeth are required to support mechanical resistance, and in them too we find the structure admirably adapted to this end, but in the teeth the mechanical force is mediate in its application; and in them we find a peculiar arrangement of osseous structure, enabling them to withstand without injury the force applied. And, as a further adaptation, they are endowed with a lower degree of organization than the bones, which, covered by soft tissues, are less susceptible of injury from mechanical force.

The teeth are said to possess in themselves no power of reproduction by which an injury can be repaired. This is not strictly true. The injury to which, in a state of nature and health, they are most liable, is wearing away of the masticating surface from use. The worn surface certainly is not renewed, but the teeth increase in density, and the pulp-cavity diminishes in size by the formation of dentine, so that the actual amount of dentine is not diminished, while the density is increased. In each of these actions we may recognise a form of renewal, which in some degree compensates for the loss by abrasion. If the whole act of mastication is, from any cause, thrown upon two or three teeth, then these naturally, by the excessive use, wear away, till at last the whole crown is exhausted.

The teeth are important as organs of articulation; so much so, that when lost we can scarcely make ourselves intelligible. The physiology of the teeth, when limited to this use, is well worth investigating, had we time at our disposal; but before leaving the subject, I may draw your attention to a few curious facts in connection with the form of the mouth in those possessed of a good voice and distinct articulation.

Wherever you have a fine, clear, sonorous voice, you will find well formed and well arranged teeth; each tooth will occupy its proper place. But, what is perhaps still more important, the hard palate will be well formed; that is, it will present a section of a large arch, perfectly free from contraction either from side to side or from before backwards. There will not be a deep vaulted form, neither will there be a sudden elevation immediately behind the front teeth, so common in those who speak with indistinctness; on the contrary, the palate will rise gradually. The mouth and its dental appendages are not of the first importance in relation to the voice, yet they are highly important as auxiliaries, and as such their condition should not be lost sight of.

In our next lecture, I shall commence with diseases of the teeth.

